# Subjective perceptions of workload and stress of emergency service personnel depending on work-related behavior and experience patterns

**DOI:** 10.1007/s10049-022-01076-y

**Published:** 2022-09-08

**Authors:** Beatrice Thielmann, Heiko Schumann, Julia Botscharow, Irina Böckelmann

**Affiliations:** Institute of Occupational Medicine, Medical Faculty, Otto-von-Guericke-University, Leipziger Str. 44, (Building 20), 39120 Magdeburg, Germany

**Keywords:** Strain, Relaxation, Mental health, Rescue service, Work setting, Belastung, Entspannung, Psychische Gesundheit, Rettungsdienst, Arbeitsumfeld

## Abstract

**Purpose:**

Stressors due to the workload in the ambulance service are numerous and can be positively counteracted by work-related behaviors and experiences. We analyzed the subjective perceptions of workload and stress as a function of work-related behavior and experience patterns among emergency service personnel (EMP).

**Methods:**

A total of 276 EMP (94.6% men) participated (average age: 39.3 ± 8.04 years). Data on the stress situations of ambulance service staff according to the Slesina questionnaire, the Recovery–Stress Questionnaire (EBF), and the Questionnaire for Physical, Psychological and Social Symptoms (KOEPS) were obtained. Participants were classified into four patterns (A, B, G, and S) based on the Work-Related Behavior and Experience Patterns (AVEM) questionnaire.

**Results:**

Overall, 32% of EMP were classified into AVEM risk patterns A and B. For half of the stress factors examined (23/46), the data were significantly different among the AVEM groups. Individuals with AVEM risk patterns have higher stress and lower recovery scores on the EBF and more physical, psychological, and social-communicative impairments shown using the KOEPS (all variables *p* < 0.001). Analyses showed moderate correlations among the AVEM dimensions (exceptions included striving for perfection, subjective importance of work, and work-related ambition), and the main scales of the EBF and KOEPS.

**Conclusion:**

Work-related patterns of behavior and experience are used to determine how stress is handled, and it is possible to distinguish between patterns that promote health and those that hazardous to it. Individuals with AVEM patterns that are a risk to their health experience high stress, low recovery, and increased physical, psychological, and social-communicative impairments. Health-promoting work-related behaviors can be used to counteract stress. Companies developing preventive health promotion measures should focus on individuals with AVEM patterns that are a risk to their health.

**Supplementary Information:**

The online version of this article (10.1007/s10049-022-01076-y) contains further tables and figures. The article and additional material are available at www.springermedizin.de. Please enter the title of the article in the search field. You will find the additional material under “Supplementary Information” at the article.

## Abstract (de)

**Hintergrund:**

Im Rettungsdienst gibt es vielfältige Belastungen, denen arbeitsbezogene Verhaltens- und Erlebensweisen als Ressource positiv entgegenwirken können. In der vorliegenden Studie wurde die subjektive Wahrnehmung arbeitsbedingter Belastungen und daraus resultierender Beanspruchung in Abhängigkeit von arbeitsbezogenen Verhaltens- und Erlebensmustern bei Einsatzkräften des Rettungsdienstes untersucht.

**Methodik:**

Teilnehmer waren 276 Einsatzkräfte (94,6 % Männer). Das Durchschnittsalter betrug 39,3 ± 8,04 Jahre. Es wurden Daten zur Belastungssituation im Rettungsdienst nach Slesina sowie aus dem Erholungs-Belastungs-Fragebogen (EBF) und den Fragebogen für körperliche, psychische und soziale Symptome (KOEPS) ermittelt. Die Probanden wurden bezüglich des arbeitsbezogenen Verhaltens- und Erlebensmusters (AVEM) in 4 verschiedene Gruppen eingeteilt und verglichen.

**Ergebnisse:**

Dabei wurden 32 % der Rettungskräfte in gesundheitsgefährdende AVEM-Muster eingestuft. Für die Hälfte der untersuchten Belastungsfaktoren (23 von 46) waren die Angaben zwischen den AVEM-Gruppen signifikant unterschiedlich. Dabei wiesen Personen mit AVEM-Risikomuster höhere Belastungen im EBF und eine niedrigere Erholung im EBF auf und gaben auch mehr körperliche, psychische und sozial-kommunikative Beeinträchtigungen im KOEPS an (alle Merkmale *p* < 0,001). Analysen ergaben überwiegend moderate Korrelationen zwischen den AVEM-Dimensionen (Ausnahmen: Streben nach Perfektion, subjektive Bedeutung von Arbeit und arbeitsbezogener Ehrgeiz) und den Hauptskalen des EBF und KOEPS.

**Schlussfolgerung:**

Mit arbeitsbezogenen Verhalten- und Erlebensmustern wird der Umgang mit Stress ermittelt, wobei es möglich ist, zwischen gesundheitsförderlichen und -gefährdenden Mustern zu unterscheiden. Personen mit gesundheitsgefährdenden AVEM-Mustern erleben eine hohe Beanspruchung, niedrige Erholung und vermehrt körperliche, psychische und sozial-kommunikative Beeinträchtigungen. Unternehmen, die präventive Gesundheitsförderungsmaßnahmen im Betrieb anbieten, sollten sich auf diese Gruppe konzentrieren.

## Brief introduction to the subject

Physical and psychological stressors in emergency medical services (EMS) are numerous. Work-related behaviors and experiences counteract workloads. Individuals can be classified into four different patterns (A, B, G, and S) based on the Work-Related Behavior and Experience Patterns (AVEM) questionnaire. Advantages of the AVEM include the examination of health-promoting behaviors as well as health-hazardous behavior and experience patterns. They can be used specifically for health promotion and prevention programs.

## Introduction and background

Stressors in emergency medical services (EMS) are numerous. Briefly, a classification can be made between physical and psychological stressors (e.g., climatic conditions, noise, decision-making pressure, emotional stakes, shift work), personal stressors (e.g., perfectionism, role ambiguity), or organization-related stressors (e.g., poor communication, bad working atmosphere; [8, 23, 28]). In addition, there are professional stressors such as a lack of respect, low chances of professional career growth, and unclear protection under the law with a high legal risk [6, 23]. There is an increasing tendency for stress in the workplace, which was highlighted by the prevalence of the SARS-CoV‑2 pandemic [24]. Depending on the type, timing, and severity of stress, as well as its cognitive evaluation, stress can have various effects on the body and trigger health complaints [20, 30], such as mental disorders [2], cardiovascular diseases [13], or the suppression of the cellular and humoral immune response [25].

The stress–strain concept describes the stress response as a mental state of stress that persists for a period of time even after the stressors (strains) have been removed. From this, it is possible to draw conclusions regarding the stress level of a person, considering that with an increasing number of strains, their compensation is at risk of being exhausted [11, 16]. Work-related stressors thus play an important role in the development of stress, but personal characteristics such as work-related behavior and experience patterns can also have a productive or counterproductive effect on the subjective perception of stress, which ultimately results in an individual’s perception of stress. People with personality traits such as agitation, aggressiveness, inhibition, and mood lability express health risk behavior and experience patterns related to their jobs significantly more often [7].

Personality diagnostics can include the recording of work-related behaviors, attitudes and habits, i.e., how a person meets work-related requirements and helps to shape them [18]. The basic health psychological concepts of work-related behavior and experience patterns (AVEM) according to Schaarschmidt and Fischer [17] include Antonovsky’s resource theory (1979), the concept of coherence experience by Antonovsky [3], and the transactional stress and coping concept of Lazarus [12].

There is limited literature that considers work-related behaviors and experiences in the context of recovery and stress and physical, psychological, and social-communicative impairments; hardly any of the literature includes emergency services personnel (EMP). The aim of this study was to examine the subjective perceptions of work-related stress and resulting stress as a function of work-related behavioral and experiential patterns among EMP. We hypothesized that emergency service personnel with AVEM patterns that are a risk to their health would have higher stress and poor recovery. Consequently, we hypothesized that they would have more physical, social-communicative, and psychological impairments.

## Study design and methods

### Participants

There were 261 men (94.6%) and 15 women (5.4%) who participated in this quantitative cross-sectional health study. The mean age was 39.3 ± 8.04 years and the age range was 23–62 years. The respondents included 70 (25.4%) EMP from the ambulance service of the professional fire department and 206 (74.6%) individuals from the ambulance service of aid organizations (e.g., German Red Cross, Worker’s Samaritan Organization).

The respondents’ participation was voluntary and anonymous, and this study was conducted in accordance with the 1975 Declaration of Helsinki in its current, revised version. Approval was obtained from the local ethics committee (registration number 61/13).

The inclusion criteria were defined as follows: individuals who had completed rescue service training and had a full-time job in the ambulance service as an emergency medical technician, paramedic, or emergency paramedic. The exclusion criteria were formulated as individuals who worked at least 20 h/week and had less than 3 years of service in the rescue service. The participants were recruited by writing to the rescue services and schools and invited by mail to participate.

A total of 850 questionnaires were distributed to EMP from aid organizations and professional fire departments. The response rate was 32.5% (*n* = 276).

### Materials

The questionnaire included sociodemographic and job-related questions as well as four standardized self-assessment questionnaires:

- Assessment of the stress situation in the rescue service according to the Slesina questionnaire [26]
- Work-Related Behavior and Experience Patterns (AVEM) questionnaire by Schaarschmidt and Fischer [19]
- The Recovery-Stress Questionnaire (EBF) according to Kallus [11]
- The Questionnaire for Physical, Psychological, and Social Symptoms (KOEPS; [14])

#### Stress situation in the ambulance service according to Slesina

The Slesina questionnaire [26] allows statements to be made about one’s own experience of stress on the basis of 47 questions. The intensity of the demands and the resulting subjective feelings of stress are determined in four areas (work content, work organization, position, and work environment factors). The formulation of the questions is presented in the supplemental materials.

#### The AVEM questionnaire by Schaarschmidt and Fischer

The AVEM questionnaire identifies work-related behavioral and experience characteristics using 11 dimensions (Table 1) from the following three domains: *engagement* with work, *resilience* in dealing with the everyday stress of work, and *emotions* associated with work and life in general [19]. The questions focus on common behaviors, attitudes, and habits in relation to one’s own working life. High values indicate a high level of expression for each dimension.

**Table 1:** Characterization of the four AVEM patterns

AVEM pattern	Characteristics
*Risk pattern*	
A	Excessive work engagement
	Limited distancing ability from work-related problems
	Reduced emotional resilience to stress
	Limited enjoyment of life
B	Reduced work engagement
	Limited distancing ability from work-related problems
	Marked tendency to resignation
	Reduced emotional resilience to stress
	Significantly limited enjoyment of life
*Healthy pattern*	
G	Clear, but not excessive work engagement
	Good distancing from work-related problems
	Proactive problem-solving
	Resilience to stress
	Positive feelings about life
S	Taking it easy
	Low level of work engagement
	Marked distancing ability from work-related problems
	Emotional resilience to stress
	Intervention from point of view of motivation can be recommended

On the basis of the levels of expression for all dimensions, the participants were classified into risk pattern (A, B) and healthy pattern groups [18]. Patterns with a “full” expression (a pattern of > 95% expression), an “accentuated” expression (a pattern between > 80% and ≤ 95% expression), or a “tendential” pattern expression (a pattern between ≥ 60 and ≤ 80%, not including a second pattern with ≥ 30% expression) were considered in this study. The normal score ranges between 4 and 6. The individual AVEM patterns differ in the 11 dimensions.

#### EBF according to Kallus

The EBF questionnaire [11] is able to assess the degree of recovery and stress of EMP and thus describes the current mental and physical state of health of a respondent during the past 3 days. In the context of this study, the short version with 25 items was used, which comprises 12 subscales (seven subscales define the main stress scale and five form the main recovery scale). High expressions of the values indicate strong stress/strain or good recovery activities.

#### KOEPS according to Manz

The KOEPS questionnaire [14] helps determine symptoms of the last 4 weeks on the basis of 60 items. Symptoms are divided into three categories—physical, psychological and social-communicative impairments—are additionally summarized in a category for total impairments. The statements are answered by means of a fourfold gradation from “did not apply” to “applied very much.” The higher the score in the evaluation, the more complaints indicated for the respective area.

### Statistical analysis

Statistical analysis was performed using the statistical program SPSS Statistics 28 (IBM, New York, USA). After testing for normal distribution, a descriptive description of the total sample was carried out. Cross tables and cross-tabulations with Pearson’s chi-square tests were carried out for the evaluations of the stress factors and their assessment. For the evaluations of the KOEPS and EBF dimensions, the Kruskal–Wallis test was used first, and if significance was found between the AVEM groups, the Mann–Whitney *U* test was applied. Finally, Spearman’s correlation analyses were performed between the AVEM dimensions and the KOEPS categories, and the total scale or the main Stress–Recovery scales and the dimensions of the EBF. For the probability of error, a value of α = 5% was assumed as the significance level in the statistical procedures.

## Results

### Occupational data

The sample of EMP from four states of the FRG (Saxony-Anhalt, Lower Saxony, Brandenburg, and Berlin) was made up of the following:

- 88.4% (*n* = 244) paramedics and emergency paramedics
- 11.6% (*n* = 32) emergency medical technicians

The average length of service of the surveyed EMP was 12.9 ± 7.54 years (range: 3–35 years). Depending on the operational area (size, location) and operational region (city, country), the average alarm operation frequency (based on 12 h) varied between 2 and 11 alerts, with a mean of 5.7 ± 1.86 alerts. The majority of respondents worked in densely to very densely populated deployment areas (*n* = 195, 70.6%), and the remainder worked in sparsely to very sparsely populated areas. The average weekly working hours were 51.44 ± 6.39 h (40–96 h/week). A total of 139 (50.4%) respondents worked over 48 h/week (over the permitted legal average working time in Germany).

### Classification of EMP into work-related behavioral and experiential patterns

It was possible to assign 205 (74.3%) of the 276 EMP to one of the four AVEM patterns with a “full” (14.5%, *N* = 40), an “accentuated” (30.4%, *N* = 84), or a “tendential” (29.4%, *N* = 81) expression pattern. The subsequent classification of the EMP respondents into AVEM patterns was possible:

- Risk pattern A: 40 (19%)
- Risk pattern B: 27 (13%)
- Risk pattern G: 63 (31%)
- Risk pattern S: 75 (37%)

A combination of characteristics from two patterns was present in 18.1% (*N* = 50) of the total sample, and no assignment to a pattern affiliation could be made for 7.6% (*N* = 21) of the EMP. The expression of the individual AVEM dimensions in participants with one of the four AVEM patterns is shown in the Supplement (see supplementary information online). “Willingness to work until exhausted” was most pronounced in those classified with AVEM risk pattern A (6.9 ± 1.37 points), which was above the normal limit. “Tendency to resignation in the face of failure” was most pronounced in those assigned to AVEM risk pattern B (5.6 ± 1.31).

Half of the stress factors studied (23 out of 46) were significantly different among the AVEM groups, e.g., physical work (*p* < 0.027), overhead work (*p* = 0.003), vibration/oscillation (*p* = 0.038), shift work (*p* = 0.049), trouble with superiors (*p* < 0.001), trouble with colleagues (*p* = 0.001), pressure to perform (*p* = 0.012), thinking (*p* < 0.001), independent division of work (*p* = 003), responsibility for machines/materials (*p* < 0.001), and chemical substances (*p* < 0.001). Therefore, the risk groups with patterns A and B had the highest values for almost all stress factors. The subjectively perceived stressors and the intensity of the assessment of these stress factors differentiated by AVEM patterns are listed in Supplemental Tables 2a, 2b, and 2c (see supplementary information online–Supplement 2a: Physical stressors; Supplement 2b: Organizational and psychological stressors; and Supplement 2c: Stressors due to work environments).

### Recovery and stress within the AVEM patterns

The examination of both the main scales of stress and recovery as well as their subscales showed highly significant differences between individuals in the AVEM A and B risk pattern groups and the AVEM G and S health pattern groups (*p* < 0.001). Here, each of the AVEM risk patterns offered the highest scores in stress and the lowest scores in recovery. The results are shown in Table 2. The EMP with AVEM risk pattern A complained most of “unresolved conflicts—lack of success” (3.1 ± 1.25), and those with risk pattern B complained of “overtiredness—time pressure” (2.8 ± 1.11). For EMP in both risk pattern groups, “success—capability” was the lowest recovery variables (1.8 ± 0.79 and 1.9 ± 0.82, respectively).

**Table 2:** Expression of stress and recovery within the AVEM patterns

Feature	AVEM sample				*p*_Kruskal—Wallis_	*p*_Mann—Whitney_
	A	B	G	S		
	AV ± SD Median (Min–Max) [95% CI]					
*EBF*						
Strain	2.5 ± 0.96 2.4 (0.2–4.9) [2.16–2.77]	2.6 ± 0.70 2.4 (1.4–4.0) [2.31–2.86]	1.3 ± 0.78 1.4 (0.1–3.5) [1.12–1.51]	1.5 ± 0.77 1.5 (0.1–4.2) [1.3–1.7]	< 0.001	A‑G*** A‑S*** B‑G*** B‑S***
General stress—despondency	2.2 ± 1.13 2 (0.5–6) [1.86–2.59]	2.6 ± 1.10 2.5 (1–5.5) [2.12–2.99]	0.9 ± 0.99 0.5 (0–4) [0.69–1.19]	1.3 ± 0.99 1 (0–5) [1.05–1.50]	< 0.001	A‑G*** A‑S*** B‑G*** B‑S***
Emotional stress	2.5 ± 1.00 2.5 (1–5.5) [2.22–2.86]	2.6 ± 0.95 2.5 (1–4) [2.18–2.93]	1.5 ± 0.89 1.5 (0–3.5) [1.23–1.68]	1.5 ± 0.89 1.5 (0–4) [1.29–1.70]	< 0.001	A‑G*** A‑S*** B‑G*** B‑S***
Social tensions	2.5 ± 1.29 2.5 (0.5–6) [2.10–2.93]	2.7 ± 1.16 2.5 (1–5) [2.28–3.20]	1.4 ± 0.95 1.5 (0–4.5) [1.12–1.60]	1.4 ± 0.90 1 (0–4) [1.15–1.56]	< 0.001	A‑G*** A‑S*** B‑G*** B‑S***
Unresolved conflicts—lack of success	3.1 ± 1.25 3 (1–6) [2.72–3.53]	2.7 ± 0.85 2.5 (1–4.5) [2.37–3.04]	1.7 ± 1.05 1.5 (0–4) [1.44–1.96]	1.9 ± 1.03 2 (0–5) [1.64–2.11]	< 0.001	A‑G*** A‑S*** B‑G*** B‑S***
Overtiredness—time pressure	2.7 ± 1.30 2.5 (0.5–5.5) [2.30–3.13]	2.8 ± 1.11 3 (0–4.5) [2.38–3.25]	1.5 ± 1.19 1.5 (0–4.5) [1.21–1.81]	1.6 ± 0.93 1.5 (0–4) [1.42–1.85]	< 0.001	A‑G*** A‑S*** B‑G*** B‑S***
Lack of energy, lack of concentration	2.3 ± 0.75 2 (1–4) [2.01–2.49]	2.5 ± 0.80 2.5 (1.5–4) [2.15–2.78]	1.4 ± 0.87 1 (0–4) [1.15–1.58]	1.6 ± 0.95 1.5 (0–4.5) [1.41–1.85]	< 0.001	A‑G*** A‑S*** B‑G*** B‑S***
Physical ailments	2.1 ± 1.00 2 (0.5–4.5) [1.77–2.41]	2.2 ± 1.11 2 (0–5) [1.75–2.62]	1.0 ± 0.83 1 (0–3.5) [0.77–1.18]	1.2 ± 0.95 1 (0–5) [0.95–1.39]	< 0.001	A‑G*** A‑S*** B‑G*** B‑S***
Recovery	2.2 ± 0.54 2.25 (0.7–3.2) [2.05–2.40]	2.3 ± 0.68 2.1 (1.2–3.9) [1.99–2.52]	3.6 ± 0.86 3.6 (1.6–5.4) [3.38–3.81]	3.2 ± 0.81 3.3 (0.9–5) [3–3.38]	< 0.001	A‑G*** A‑S*** B‑G*** B‑S***
Success—capability	1.8 ± 0.79 1.75 (0.5–4.5) [1.59–2.09]	1.9 ± 0.82 1.5 (0.5–3.5) [1.53–2.18]	2.8 ± 1.15 2.5 (0–5.5) [2.52–3.10]	2.3 ± 0.98 2.5 (0–4) [2.13–2.54]	< 0.001	A‑G*** A‑S** B‑G*** B‑S*
Recovery in the social field	2.1 ± 0.81 2 (0.5–4) [1.86–2.39]	2.2 ± 1.07 2 (0.5–4.5) [1.82–2.66]	3.3 ± 1.32 3.5 (0.5–6) [2.98–3.65]	3.0 ± 1.16 3 (0.5–5.5) [2.74–3.27]	< 0.001	A‑G*** A‑S*** B‑G*** B‑S**
Physical recovery	2.3 ± 0.76 2.25 (1–4) [2.03–2.52]	2.3 ± 0.82 2.5 (1–4) [2.01–2.66]	3.7 ± 1.04 4 (1.5–5.5) [3.43–3.95]	3.2 ± 1.07 3 (0.5–5.5) [2.95–3.44]	< 0.001	A‑G*** A‑S*** B‑G*** B‑S***
General recovery, wellbeing	2.4 ± 0.79 2.5 (1–5) [2.18–2.69]	2.4 ± 0.89 2.5 (1–4) [2.04–2.74]	4.2 ± 1.05 4.5 (1–6) [3.89–4.42]	3.6 ± 0.97 3.5 (1–5.5) [3.36–3.80]	< 0.001	A‑G*** A‑S*** B‑G*** B‑S***
Restorative sleep	2.5 ± 1.07 2.5 (0–4.5) [2.12–2.80]	2.5 ± 0.81 2.5 (1–4) [2.14–2.78]	4.0 ± 1.40 4 (0.5–6) [3.63–4.34]	3.8 ± 1.26 4 (0.5–6) [3.53–4.11]	< 0.001	A‑G*** A‑S*** B‑G*** B‑S***

*CI* confidence interval, *AV* *±* *SD* average value and standard deviation

* *p* < 0.05, ** *p* < 0.01, *** *p* < 0.001

### Physical, psychological, and social-communicative impairments of the AVEM patterns

The EMP assigned to the AVEM risk patterns had significantly higher physical, psychological, and social-communicative impairments than those assigned to the AVEM health-promoting patterns (*p* < 0.001). Consequently, total impairments were also significantly higher in the EMP assigned to the AVEM risk patterns than in those assigned to the AVEM health-promoting patterns. The results are shown in Supplement 3 (see supplementary information online).

### Relationships of AVEM dimensions with total impairments (KOEPS) and stress and recovery (EBF)

**Fig. 1:**
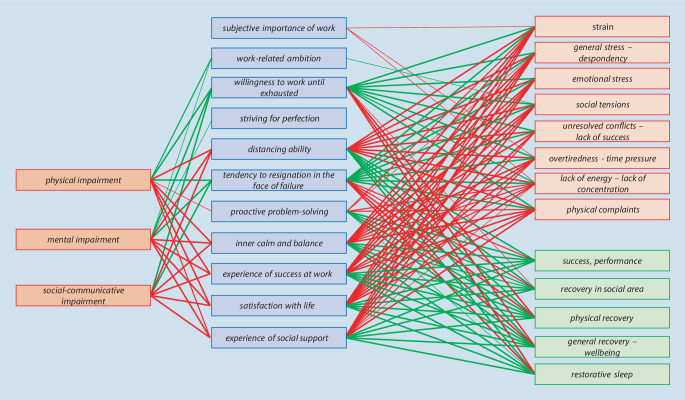
Overview of correlations between AVEM dimensions and KOEPS or EBF dimensions

Figure 1 show that certain AVEM dimensions correlated very strongly with the physical, psychological, and social-communicative impairments of the KOEPS or with strain or recovery, but the others did not (e.g., “striving for perfection,” “work-related ambition,” or “subjective importance of work”). In Fig. 1, positive correlations are shown in green and negative correlations in red; the thicker the line, the stronger the correlation.

The “satisfaction with life” dimension of the AVEM was strongly positively correlated with the “recovery” dimension of the EBF (ρ = 0.532) and was moderately negatively correlated with the “general stress—despondency” dimension of the EBF (ρ = −0.468). The distancing ability dimension of the AVEM was also almost strongly positively correlated with the “recovery” dimension of the EBF (ρ = 0.498). Correlations of the total KOEPS scale and the main EBF strain and recovery variables with the AVEM dimensions are shown in Supplement 4 (see supplemental information online).

The AVEM dimensions “willingness to work until exhausted” and “engagement with work” of the main scale were only correlated with the EBF main scales of stress and recovery. The AVEM dimension “resilience in dealing with the everyday stress of work” of the main scale showed moderate to nearly high correlations. “Work-related ambition” was only weakly correlated with the KOEPS “total impairments” dimension. “Distancing ability” was moderately to highly correlated with the “recovery” EBF dimension, moderately negatively correlated with the “total impairments” dimension of the KOPS, and positively correlated with the “stress” dimension of the EBF. All AVEM dimensions of the main scale “emotions associated with work and with life in general” were moderately negatively correlated with the “total impairment” dimension of the KOEPS and the “stress” dimension of the EBF and was positively correlated with the “recovery” dimension of the EBF.

## Discussion

This study focused on the subjective perceptions of stress and strain of full-time EMP as a function of their work-related behavioral and experience patterns that can be classified as either healthy or risky. Work-related behavioral and experiential patterns include “engagement with work,” “resilience in dealing with the everyday stress of work,” and “emotions associated with work and life in general” [17]. They are considered components of mental health and personality traits [5]. Although the stressful situations in EMS experienced by the study respondents were assumed to be comparable, the subjective information on the occurrence of the stressors and the intensity of exposure to stressors in the workplace varied greatly depending on an individual’s work-related stress and experience patterns. Of the 276 participating EMP, 205 were assigned to one of the AVEM patterns. The majority of the rescue workers had healthy patterns (G and S), and 32% had AVEM risk patterns (A and B) that were hazardous to their health. This were slightly fewer subjects than policemen in another study, 40% of whom had health-hazardous AVEM risk patterns [4]. Comparatively, further studies with individuals who do not work in operative task forces found that 42% (27% A and 15% B) of teachers and 69% (39% A and 30% B) of medical students [1] had AVEM risk patterns.

Typical stress factors were identified for the EMP and compared among the four AVEM patterns. Individuals with AVEM risk patterns (A and B) assessed stress factors more frequently and felt more stressed by them than those with healthy patterns (G and S). This was the case for the physical stress factors (e.g., physical work, overhead work), organizational and psychological factors (e.g., trouble with superiors or colleagues, pressure of decision-making), and environmental factors during work (e.g., chemical substances). Overall, the frequencies of work-related environmental stress factors were the least distinguishable among the AVEM groups. Comparable studies are not known. These differences can be explained by the different experiences of self-esteem impairment, performance insufficiency, and exhaustion of individuals in the four AVEM patterns [17]; presumably, stressors are perceived more strongly. It also appears that attitude toward life as a whole can play a large part in the results. The attitude toward life of individuals classified with AVEM risk pattern B was the worst, followed by that of individuals classified with risk pattern A.

A limiting factor here is that life events, such as caring for relatives and life events, were not taken into account. The data were also collected before the current SARS-CoV‑2 pandemic and this was also not taken into account; this would be an interesting research topic. It also cannot be ruled out with certainty whether strongly stressed EMP in the ambulance service participated in this study. Stress in the work context of healthcare workers and thus also general stress tended to increase in the course of the pandemic, e.g., due to increased hygiene measures and concern about infections [24, 27]. A study from Denmark showed longer operation times in prehospital operations and transport to the hospital, although the frequency of alarm operations decreased at the beginning of the pandemic [9]. All of this can have an additional impact on recovery and stress. The differences in AVEM patterns could also be reflected in the stress–recovery experiences of the EMP. Individuals classified with AVEM risk patterns A and B had higher stress and poorer recovery. For those classified with AVEM risk pattern A, the dimension “unsolved conflicts/lack of success” was the most pronounced, and for those with risk pattern B, “fatigue/time pressure” was the most pronounced. In terms of recovery, “success/performance” was the lowest for both AVEM risk pattern groups.

Existing recovery activities and, above all, subjectively perceived recreational opportunities are undoubtedly important health indicators because they are a way of compensating for stress [11]. In our study, the stress factor “shift work” was assessed significantly differently by the four AVEM groups; the stress caused by this factor was rated to be “often” in 85% of the EMP with risk pattern A and in 66.7% of the EMP with risk pattern B. The study by Schmid et al. showed that among EMP, overtiredness was highest after night shifts, and the ability to recover based on the EBF was significantly lower after night shifts than after day shifts or leisure time. Likewise, somatic complaints were significantly more frequent after night duty than after leisure time [22]. Similar findings can be suggested from our data. Individuals with AVEM risk patterns A and B complained about more stress factors and felt more stressed without sufficient rest, which was also reflected in their physical, psychological, and social-communicative impairments. Here, those with AVEM risk patterns A and B also indicate having more impairments. Another limitation is that the study did not explicitly examine sleep disturbances. Some people are genetically unable to get up frequently at night, which could explain some of the results [21].

In summary, the AVEM main scale of “engagement with work” was only slightly correlated with the “stress” dimension of the EBF and the overall impairments dimension of the KOEPS. The AVEM scales of “resilience in dealing with the everyday stresses of work” and “emotions associated with work and life in general” showed moderate-to-high correlations with the previously mentioned scales of the KOEPS and EBF.

The data demonstrate a need for action, especially for EMP who have risky AVEM patterns. In particular, objective methods for determining stress are recommended for these employees, e.g., by the monitoring of heart rate variability, which can be derived by means of long-term electrocardiograms [29]. Preventive courses to strengthen resilience could be helpful, for example, to act in a problem-oriented manner [15]. Training and competence development could also create more professional autonomy, which can have a positive effect on stress [10].

## Conclusion

The subjective perceived stress levels of individuals working in emergency services are partly high. Work-related behavior and experience patterns determine how stress is handled, and it is possible to distinguish between health-promoting and health-hazardous patterns. Individuals classified with risky AVEM patterns had high stress, low recovery, and increased physical, psychological, and social-communicative impairments in our study. We recommend offering preventive health promotion interventions among emergency service personnel depending on the AVEM pattern.

## Supplementary Information

ESM 1: Expression of AVEM dimensions in respondents with different AVEM pattern

ESM 2: Stress factors and their evaluation

ESM 3: Physical, psychological, and social-communicative impairments within the AVEM groups

ESM 4: Correlation analysis according to Spearman of the AVEM dimensions with the main scales of the KOEPS and EBF

## Declarations

All investigations on humans were carried out in accordance with the appropriate ethics committee, in accordance with national legislation as well as according to the declaration of Helsinki. All informed participants submitted a declaration of consent. The study was authorized by Otto von Guericke University in Magdeburg, Germany (register no. 61/13).
