# Supplementary material for: Subjective perceptions of workload and stress of emergency service personnel depending on work-related behavior and experience patterns
Source: Notf Rett Med. 2022 Sep 8;25(Suppl 2):15–22. doi: 10.1007/s10049-022-01076-y (PMC9454386; doi:10.1007/s10049-022-01076-y)
Supplement: Supplementary file 1 — ESM 1: Expression of AVEM dimensions in respondents with different AVEM pattern [file 10049_2022_1076_MOESM1_ESM.pdf]

Supplementary material to the article "**Subjective perceptions of workload and stress of emergency service personnel depending on work-related behavior and experience patterns**" by Beatrice Thielmann, Heiko Schumann, Julia Botscharow and Irina Böckelmann (2022) in *Notfall+Rettungsmdedizin*.
Article and supplementary material are available at www.springermedizin.de. Please enter the article title in the search there.

Expression of AVEM dimensions in respondents with different AVEM pattern.

**Feature**	**AVEM sample**			
	A<br>(n = 40)	B<br>(n = 27)	G<br>(n = 63)	S<br>(n = 75)
	AV ± SD<br>Median (Min - Max)<br>[95% - CI]			
**Subjective importance of work**	6,1 ± 1,54<br>6 (2 - 9)<br>[5,56 - 6,54]	4,0 ± 1,18<br>4 (1 - 5)<br>[3,53 - 4,47]	5,0 ± 1,63<br>5 (2 - 9)<br>[4,56 - 5,38]	3,4 ± 1,39<br>3 (1 - 7)<br>[3,11 - 3,75]
**Work-related ambition**	6,7 ± 1,60<br>7 (3 - 9)<br>[6,19 - 7,21]	4,5 ± 1,28<br>5 (2 - 7)<br>[4.01 - 5,03]	6,8 ± 1,31<br>7 (4 - 9)<br>[6,49 - 7,16]	4,0 ± 1,27<br>4 (1 - 6)<br>[3,69 - 4,28]
**Willingness to work until exhausted**	6,9 ± 1,37<br>6 (4 - 9)<br>[6,41 - 7,29]	5,2 ± 1,31<br>5 (3 - 9)<br>[4,70 - 5,74]	5,3 ± 1,49<br>5 (1 - 9)<br>[4,88 - 5,63]	3,8 ± 1,35<br>4 (1 - 6)<br>[3,49 - 4,11]
**Striving for perfection**	5,8 ± 1,42<br>6 (2 - 9)<br>[5,32 - 6,23]	4,2 ± 1,41<br>4 (2 - 7)<br>[3,59 - 4,70]	2,8 ± 1,33<br>6 (3 - 9)<br>[5,41 - 6,08]	3,8 ± 1,11<br>4 (1 - 6)<br>[3,50 - 4,02]
**Distancing ability**	4,3 ± 1,31<br>4 (1 - 7)<br>[3,91 - 4,74]	5,3 ± 1,16<br>5 (3 - 7)<br>[4,80 - 5,72]	6,9 ± 1,12<br>7 (4 - 9)<br>[6,61 - 7,17]	7,4 ± 0,79<br>7 (6 - 9)<br>[7,19 - 7,55]
**Tendency to resignation in the face of failure**	5,6 ± 1,28<br>6 (3 - 8)<br>[5,19 - 6,01]	5,6 ± 1,31<br>6 (2 - 8)<br>[5,11 - 6,15]	3,6 ± 1,63<br>4 (1 - 8)<br>[3,18 - 4,00]	3,2 ± 1,28<br>3 (1 - 6)<br>[2,94 - 3,54]
**Proactive problem-solving**	4,9 ± 1,74<br>5 (1 - 8)<br>[4,34 - 5,46]	3,3 ± 1,40<br>3 (1 - 6)<br>[2,70 - 3,81]	6,1 ± 1,53<br>6 (2 - 9)<br>[5,71 - 6,48]	3,6 ± 1,47<br>3 (1 - 8)<br>[3,29 - 3,96]
**Inner calm and balance**	4,6 ± 1,08<br>4,5(2 - 7)<br>[4,23 - 4,92]	4,0 ± 1,11<br>4 (2 - 6)<br>[3,56 - 4,44]	6,7 ± 1,36<br>7 (4 - 9)<br>[6,36 - 7,04]	5,7 ± 1,49<br>6 (2 - 9)<br>[5,36 - 6,05]
**Experience of success at work**	3,4 ± 1,26<br>3 (1 - 7)<br>[3,00 - 3,80]	2,0 ± 1,00<br>2 (1 - 4)<br>[1,60 - 2,40]	4,9 ± 1,49<br>5 (1 - 8)<br>[4,48 - 5,23]	4,1 ± 1,53<br>4 (1 - 8)<br>[3,73 - 4,43]
**Satisfaction with life**	3,1 ± 1,44<br>3 (1 - 6)<br>[2,67 - 3,58]	1,9 ± 1,00<br>2 (1 - 5)<br>[1,53 - 2,32]	5,3 ± 1,28<br>5 (2 - 8)<br>[5,01 - 5,66]	4,5 ± 1,17<br>4 (1 - 7)<br>[4,22 - 4,76]
**Experience of social support**	3,4 ± 1,22<br>3 (1 - 6)<br>[3,01 - 3,79]	3,2 ± 1,25<br>3 (1 - 6)<br>[2,73 - 3,72]	5,9 ± 1,46<br>6 (3 - 8)<br>[5,52 - 6,26]	5,0 ± 1,56<br>5 (2 - 8)<br>[4,60 - 5,32]

Notes. Since the classification of the AVEM patterns is based on the expression of the dimensions, a significance of $p<0.001$ has been calculated in the Kruskal-Wallis test in each dimension. CI=confidence interval, AV ± SD= average valvue and standard deviation
