# Supplementary material for: Subjective perceptions of workload and stress of emergency service personnel depending on work-related behavior and experience patterns
Source: Notf Rett Med. 2022 Sep 8;25(Suppl 2):15–22. doi: 10.1007/s10049-022-01076-y (PMC9454386; doi:10.1007/s10049-022-01076-y)
Supplement: Supplementary file 2 — ESM 2: Stress factors and their evaluation [file 10049_2022_1076_MOESM2_ESM.pdf]

Supplementary material to the article "**Subjective perceptions of workload and stress of emergency service personnel depending on work-related behavior and experience patterns**" by Beatrice Thielmann, Heiko Schumann, Julia Botscharow and Irina Böckelmann (2022) in *Notfall+Rettungsmedizin*.
Article and supplementary material are available at www.springermedizin.de. Please enter the article title in the search there.

2a. Most frequent physical stress factors and their evaluation

	"How often or how strongly does this characteristic or factor apply to your work?"					p_{χ^2} Pearson	"Does this make you feel physically or mentally stressed or strained?"					p_{χ^2} Pearson
stress factor		A	B	G	S			A	B	G	S	
inappropriate posture	*never*	0(0,0%)	0(0,0%)	1(1,6%)	0(0,0%)	0,492	*no*	8(20,0%)	4(14,8%)	17(27,0%)	26(34,7%)	0,255
	rarely	6(15,0%)	6(22,2%)	13(20,6%)	18(24,0%)		*yes*	32(80,0%)	23(85,2%)	46(73,0%)	41(65,3%)	
	moderately	17(42,5%)	5(18,5%)	28(44,4%)	31(41,3%)							
	often	17(42,5%)	16(59,3%)	21(33,3%)	26(34,7%)							
lifting heavy loads	*never*	0(0,00%)	0(0,0%)	1(1,6%)	0(0,0%)	0,959	*no*	9(22,5%)	4(14,8%)	21(33,3%)	24(32,0%)	0,241
	rarely	7(17,5%)	5(18,5%)	14(22,2%)	15(20,0%)		*yes*	31(77,5%)	23(85,2%)	42(66,7%)	51(68,0%)	
	moderately	20(50,0%)	13(48,1%)	34(54,0%)	37(49,3%)							
	often	13(32,5%)	9(33,3%)	14(22,2%)	23(30,7%)							
carrying heavy loads	*never*	0(0,0%)	0(0,0%)	2(3,2%)	1(1,3%)	0,949	*no*	8(20,0%)	4(14,8%)	21(33,3%)	26(34,7%)	**0,047**
	rarely	6(15,0%)	6(22,2%)	12(19,0%)	13(17,3%)		*yes*	32(80,0%)	23(85,2%)	42(66,7%)	49(65,3%)	
	moderately	19(47,5%)	14(51,9%)	31(49,2%)	39(52,0%)							
	often	15(37,5%)	7(25,9%)	18(28,6%)	22(29,3%)							
physical work	*never*					**0,027**	*no*	9(22,5%)	5(18,5%)	23(36,5%)	34(45,3%)	**0,044**
	rarely	1(2,5%)	4(14,8%)	4(6,3%)	12(16,0%)		*yes*	31(77,5%)	22(81,5%)	40(63,5%)	41(54,7%)	
	moderately	25(62,5%)	11(40,7%)	47(74,6%)	47(62,5%)							
	often	14(35,0%)	12(44,4%)	12(19,0%)	16(21,3%)							
holding heavy loads	*never*	1(2,5%)	0(0,0%)	3(4,8%)	3(4,0%)	0,923	*no*	10(25,0%)	8(29,6%)	26(41,3%)	37(49,3%)	0,075
	rarely	9(22,5%)	8(29,6%)	18(28,6%)	23(30,7%)		*yes*	30(75,0%)	19(70,4%)	37(58,7%)	38(50,7%)	
	moderately	17(42,5%)	12(44,4%)	28(44,4%)	36(48,0%)							
	often	13(32,5%)	14(22,2%)	14(22,2%)	13(17,3%)							
pulling/pushing heavy loads	*never*	0(0,0%)	0(0,0%)	5(7,9%)	4(5,3%)	0,576	*no*	11(27,5%)	7(25,9%)	30(47,6%)	41(54,7%)	**0,008**
	rarely	11(27,5%)	9(33,3%)	20(31,7%)	23(30,7%)		*yes*	29(72,5%)	20(74,1%)	33(52,4%)	34(45,3%)	
	moderately	17(42,5%)	10(37,0%)	26(41,3%)	34(45,3%)							
	often	12 30,0%)	8(29,6%)	12(19,0%)	14(18,7%)							
forced posture	*never*	1(2,5%)	1(3,7%)	10(15,9%)	6(8,0%)	0,169	*no*	17(42,5%)	11(40,7%)	38(60,3%)	47(62,7%)	0,115
	rarely	21(52,5%)	13(48,1%)	26(41,3%)	38(50,7%)		*yes*	23(57,5%)	16(59,3%)	25(39,7%)	28(37,7%)	
	moderately	14(35,0%)	12(44,4%)	17(27,0%)	27(36,0%)							
	often	4(10,0%)	1(3,7%)	10(15,9%)	4(5,3%)							
lack of exercise	*never*	2(5,0%)	0(0,0%)	6(9,5%)	8(10,7%)	**0,043**	*no*	21(52,5%)	10(37,0%)	38(60,3%)	54(72,0%)	**0,010**
	rarely	18(45,0%)	7(25,9%)	30(47,6%)	37(49,3%)		*yes*	19(47,5%)	17(63,0%)	25(39,7%)	21(28,0%)	
	moderately	17(42,5%)	17(63,0%)	16(25,4%)	21(28,0%)							

	often	3(7,5%)	3(11,1%)	11(17,5%)	9(12,0%)							
overhead work	*never*	2(5,0%)	3(11,1%)	18(28,6%)	15(20,0%)	**0,003**	*no*	21(52,5%)	15(55,6%)	54(85,7%)	60(80,0%)	**<0,001**
	rarely	31(77,5%)	14(51,9%)	33(52,4%)	50(66,7%)		*yes*	19(47,5%)	12(44,4%)	9(14,3%)	15(20,0%)	
	moderately	4(10,0%)	10(37,0%)	11(17,5%)	9(12,0%)							
	often	3(7,5%)	0(0,0%)	1(1,6%)	1(1,3%)							
standing	*never*	0(0,0%)	0(0,0%)	2(3,2%)	1(1,3%)	0,203	*no*	26(65,0%)	18(66,7%)	49(77,8%)	66(88,0%)	**0,016**
	rarely	8(20,0%)	11(40,7%)	15(23,8%)	18(24,0%)		*yes*	14(35,0%)	9(33,3%)	14(22,2%)	9(12,0%)	
	moderately	22(55,0%)	8(29,6%)	35(55,6%)	45(60,0%)							
	often	10(25,0%)	8(29,6%)	11(17,5%)	11(14,7%)							
sitting	*never*	0(0,0%)	0(0,0%)	1(1,6%)	3(4,0%)	0,536	*no*	29(72,5%)	20(74,1%)	51(81,0%)	66(88,0%)	**0,180**
	rarely	11(27,5%)	8(29,6%)	12(19,0%)	16(21,3%)		*yes*	11(27,5%)	7(25,9%)	12(19,0%)	9(12,0%)	
	moderately	21(52,5%)	16(59,3%)	36(57,1%)	45(60,0%)							
	often	8(20,0%)	3(11,1%)	14(22,2%)	11(14,7%)							
hand dexterity	*never*	0(0,0%)	0(0,0%)	1(1,6%)	2(2,7%)	0,200	*no*	28(70,0%)	21(77,8%)	51(81,0%)	67(89,3%)	0,050
	rarely	10(25,0%)	10(37,0%)	9(14,3%)	10(13,3%)		*yes*	12(30,0%)	6(22,2%)	12(19,0%)	8(10,7%)	
	moderately	16(40,0%)	12(44,4%)	17(42,9%)	41(54,7%)							
	often	12(35,0%)	5(18,5%)	26(41,3%)	22(29,3%)							
vibrations, oscillation	*never*	3(7,5%)	2(7,4%)	21(33,3%)	14(18,7%)	**0,038**	*no*	33(82,5%)	21(77,8%)	53(84,1%)	69(92,0%)	0,211
	rarely	24(60,0%)	15(55,6%)	23(36,5%)	41(54,7%)		*yes*	7(17,5%)	6(22,2%)	10(15,9%)	6(8,0%)	
	moderately	12(30,0%)	6(22,2%)	15(23,8%)	13(17,3%)							
	often	1(2,5%)	4(14,8%)	4(6,3%)	7(9,3%)							
walking	*never*	0(0,0%)	1(3,7%)	2(3,2%)	2(2,7%)	**0,032**	*no*	34(85,0%)	24(88,9%)	56(88,9%)	69(92,0%)	0,582
	rarely	16(40,0%)	9(33,3%)	6(9,5%)	14(18,7%)		*yes*	6(15,0%)	3(11,1%)	7(11,1%)	5(6,7%)	
	moderately	11(27,5%)	12(44,4%)	40(63,5%)	40(53,3%)							
	often	13(32,5%)	5(18,5%)	15(23,8%)	19(25,3%)							

2b: Most frequent organizational and psychological stress factors and their evaluation

stress factor	"How often or how strongly does this characteristic or factor apply to your work?"	A	B	G	S	p_{χ^2} Pearson	"Does this make you feel physically or mentally stressed or strained?"	A	B	G	S	p_{χ^2} Pearson
shift work	*never*	0(0,0%)	0(0,0%)	2(3,2%)	3(4,0%)	**0,049**	*no*	10(25,0%)	6(22,2%)	28(44,4%)	37(49,3%)	**0,029**
	rarely	0(0,0%)	0(0,0%)	6(9,5%)	1(1,3%)		*yes*	30(75,0%)	21(77,8%)	35(55,6%)	38(50,7%)	
	moderately	6(15,0%)	9(33,3%)	8(12,7%)	14(18,7%)							
	often	34(85,0%)	18(66,7%)	47(74,6%)	57(76,0%)							
deadline pressure	*never*	1(2,5%)	1(3,7%)	11(17,5%)	10(13,3%)	0,127	*no*	21(52,5%)	21(77,8%)	46(73,0%)	58(77,3%)	0,065
	rarely	16(40,0%)	17(63,0%)	20(31,7%)	28(37,3%)		*yes*	19(47,5%)	6(22,2%)	17(27,0%)	17(22,7%)	
	moderately	13(32,5%)	7(25,9%)	20(31,7%)	22(29,3%)							
	often	10(25,0%)	2(7,4%)	12(19,0%)	15(20,0%)							
work on weekends	*never*					**0,042**	*no*	9(22,5%)	4(14,8%)	29(46,0%)	41(54,7%)	**0,001**
	rarely	1(2,5%)	2(7,4%)	5(7,9%)	9(12,0%)		*yes*	31(77,5%)	23(85,2%)	34(54,0%)	34(45,3%)	
	moderately	11(27,5%)	13(48,1%)	27(42,9%)	35(46,7%)							
	often	28(70,0%)	12(44,4%)	31(49,2%)	31(41,3%)							
overtime	*never*	0(0,0%)	0(0,0%)	2(3,2%)	0(0,0%)	0,109	*no*	8(20,0%)	7(25,9%)	31(49,2%)	41(54,7%)	**0,002**
	rarely	8(20,0%)	8(29,6%)	24(38,1%)	30(40,0%)		*yes*	32(80,0%)	20(74,1%)	32(50,8%)	34(45,3%)	
	moderately	15(37,5%)	12(44,4%)	28(44,4%)	30(40,0%)							
	often	17(42,5%)	7(25,9%)	9(14,3%)	15(20,0%)							
trouble with superiors	*never*	7(17,5%)	0(0,0%)	20(31,7%)	7(9,3%)	**<0,001**	*no*	12(30,0%)	4(14,8%)	36(57,1%)	53(70,7%)	**<0,001**
	rarely	19(47,5%)	10(37,0%)	32(50,8%)	58(77,3%)		*yes*	28(70,0%)	23(85,2%)	27(42,9%)	22(29,3%)	
	moderately	9(22,5%)	13(48,1%)	10(15,9%)	8(10,7%)							
	often	5(12,5%)	4(14,8%)	1(1,6%)	1(1,6%)							
trouble with colleagues	*never*	5(12,5%)	0(0,0%)	15(23,8%)	7(9,3%)	**0,001**	*no*	12(30,0%)	6(22,2%)	38(60,3%)	54(72,0%)	**<0,001**
	rarely	20(50,0%)	16(59,3%)	37(58,7%)	59(78,7%)		*yes*	28(70,0%)	21(77,8%)	25(39,7%)	21(28,0%)	
	moderately	11(27,5%)	10(37,0%)	10(15,9%)	7(9,3%)							
	often	4(10,0%)	1(3,7%)	1(1,6%)	2(2,7%)							
time pressure	*never*	1(2,5%)	0(0,0%)	0(0,0%)	1(1,3%)	0,777	*no*	17(42,5%)	16(59,3%)	37(58,7%)	46(61,3%)	0,241
	rarely	9(22,5%)	9(33,3%)	14 22,2%)	14(18,7%)		*yes*	23(57,5%)	11(40,7%)	26(41,3%)	29(38,7%)	
	moderately	15(37,5%)	12(44,4%)	29(46,0%)	34(45,3%)							
	often	15(37,5%)	6(22,2%)	20(31,7%)	26(34,7%)							
responsibility for safety, health of others	*never*					**0,047**	*no*	15(10,9%)	11(40,7%)	45(71,4%)	54(72,0%)	**<0,001**
	rarely	3(7,5%)	7(25,9%)	6(9,5%)	7(9,3%)		*yes*	25(62,5%)	16(59,3%)	18(28,6%)	21(20,0%)	
	moderately	13(32,5%)	12(44,4%)	15(23,8%)	28(37,3%)							
	often	24(60,0%)	8(29,6%)	42(66,7%)	40(53,3%)							
performance pressure	*never*	1(2,5%)	0(0,0%)	5(7,9%)	9(12,0%)	**0,012**	*no*	14(35,0%)	15(55,6%)	46(73,0%)	59(78,7%)	**<0,001**
	rarely	7(17,5%)	10(37,0%)	22(34,9%)	26(34,7%)		*yes*	26(65,0%)	12(44,4%)	17(27,0%)	16(21,3%)	
	moderately	19(47,5%)	16(59,3%)	30(47,6%)	30(40,0%)							
	often	13(32,5%)	1(3,7%)	6(9,5%)	10(13,3%)							

control by superiors	*never*	2(5,0%)	0(0,0%)	13(20,6%)	5(6,7%)	**0,030**	*no*	19(47,5%)	11(40,7%)	47(74,6%)	62(82,7%)	**<0,001**
	rarely	19(47,5%)	13(48,1%)	32(50,8%)	46(61,3%)		*yes*	21(52,5%)	16(59,3%)	16(22,4%)	13(17,3%)	
	moderately	16(40,0%)	12(44,4%)	16(25,4%)	19(25,3%)							
	often	3(7,5%)	2(7,4%)	2(3,2%)	5(6,7%)							
concentration	*never*	0(0,0%)	0(0,0%)	0(0,0%)	2(2,7%)	**0,005**	*no*	24(60,0%)	18(66,7%)	45(71,4%)	56(74,7%)	0,551
	rarely	10(25,0%)	8(29,6%)	7(11,1%)	16(8,0%)		*yes*	16(40,0%)	9(33,3%)	18(28,6%)	19(25,3%)	
	moderately	12(30,0%)	12(44,4%)	19(30,2%)	42(56,0%)							
	often	18(45,0%)	7(25,9%)	37(58,7%)	25(33,3%)							
independent decision making	*never*					**0,024**	*no*	21(52,5%)	16(59,3%)	45(71,4%)	64(85,3%)	**0,003**
	rarely	5(12,5%)	7(25,9%)	2(3,2%)	9(12,0%)		*yes*	19(47,5%)	11(40,7%)	18(28,6%)	11(14,7%)	
	moderately	18(45,0%)	15(55,6%)	25(39,7%)	32(42,7%)							
	often	17(42,5%)	5(18,5%)	36(57,1%)	34(45,3%)							
contemplating	*never*	0(0,0%)	0(0,0%)	0(0,0%)	1(1,3%)	**<0,001**	*no*	21(52,5%)	20(74,1%)	43(68,3%)	63(84,0%)	**0,007**
	rarely	5(12,5%)	6(22,2%)	1(1,6%)	2(2,7%)		*yes*	19(47,5%)	7(25,9%)	20(31,7%)	12(16,0%)	
	moderately	13(32,5%)	13(48,1%)	21(33,3%)	41(54,7%)							
	often	22(55,0%)	8(29,6%)	41(65,1%)	31(41,3%)							
interruptions by superiors	*never*	1(2,5%)	1(3,7%)	12(19,0%)	12(16,0%)	0,083	*no*	20(50,0%)	14(51,9%)	51(81,0%)	68(90,7%)	**<0,001**
	rarely	22(55,0%)	18(66,7%)	42(66,7%)	47(62,7%)		*yes*	20(50,0%)	13(48,1%)	12(19,0%)	7(9,3%)	
	moderately	16(40,0%)	7(25,9%)	9(14,3%)	14(18,7%)							
	often	1(2,5%)	1(3,7%)	0(0,0%)	2(2,7%)							
independent distribution of labor	*never*	0(0,0%)	0(0,0%)	1(1,6%)	4(5,3%)	**0,003**	*no*	27(67,5%)	21(77,8%)	49(77,8%)	69(92,0%)	**0,021**
	rarely	13(32,5%)	13(48,1%)	5(7,9%)	19(25,3%)		*yes*	13(32,5%)	6(22,2%)	14(22,2%)	6(8,0%)	
	moderately	14(35,0%)	8(29,6%)	27(42,9%)	19(25,3%)							
	often	13(32,5%)	6(22,2%)	30(47,6%)	33(44,0%)							
interruptions from colleagues	*never*	2(5,0%)	2(7,4%)	8(12,7%)	6(8,0%)	0,159	*no*	33(82,5%)	24(88,9%)	54(85,7%)	69(92,0%)	0,487
	rarely	24(60,0%)	20(74,1%)	44(69,8%)	50(66,7%)		*yes*	7(17,5%)	3(11,1%)	9(14,3%)	6(8,0%)	
	moderately	12(30,0%)	5(18,5%)	11(17,5%)	19(25,3%)							
	often	2(5,0%)	0(0,0%)	0(0,0%)	0(0,0%)							
interruptions due to machine malfunctions	*never*	7(17,5%)	6(22,2%)	22(34,9%)	32(42,7%)	0,124	*no*	34(85,0%)	24(88,9%)	54(85,7%)	71(94,7%)	0,159
	rarely	28(70,0%)	20(74,1%)	35(55,6%)	39(52,0%)		*yes*	6(15,0%)	3(11,1%)	9(14,3%)	4(5,3%)	
	moderately	5(12,5%)	1(3,7%)	6(9,5%)	3(4,0%)							
	often	0(0,0%)	0(0,0%)	0(0,0%)	1(1,3%)							
monotonous work	*never*	3(7,5%)	2(7,4%)	13(20,6%)	14(18,7%)	0,375	*no*	31(77,5%)	23(85,2%)	52(82,5%)	65(86,7%)	0,771
	rarely	24(60,0%)	16(59,3%)	34(54,0%)	42(56,0%)		*yes*	9(22,5%)	4(14,8%)	11(17,5%)	10(13,3%)	
	moderately	11(27,5%)	8(29,6%)	11(17,5%)	17(22,7%)							
	often	2(5,0%)	1(3,7%)	5(7,9%)	2(2,7%)							
clock-bound work	*never*	6(15,0%)	6(22,2%)	12(19,0%)	24(32,0%)	**0,003**	*no*	37(92,5%)	24(88,9%)	55(87,3%)	68(90,7%)	0,838
	rarely	18(45,0%)	15(55,6%)	18(28,6%)	28(37,3%)		*yes*	3(7,5%)	3(11,1%)	8(12,7%)	7(9,3%)	
	moderately	14(35,0%)	4(14,8%)	18(28,6%)	17(22,7%)							
	often	2(5,0%)	2(7,4%)	15(23,8%)	6(8,0%)							
precise observation	*never*	0(0,0%)	1(3,7%)	2(3,2%)	2(2,7%)	0,439	*no*	27(67,5%)	20(74,1%)	52(82,5%)	61(81,3%)	0,216
	rarely	13(32,5%)	9(33,3%)	10(15,9%)	20(26,7%)		*yes*	13(32,5%)	7(25,9%)	11(17,5%)	14(18,7%)	
	moderately	18(45,0%)	10(37,0%)	27(42,9%)	33(44,0%)							

	often	9(22,5%)	7(25,9%)	24(38,1%)	20(26,7%)							
dependence on colleagues´ speed	*never*<br>*rarely*<br>*moderately*<br>*often*	0(0,0%)<br>16(40,0%)<br>19(47,5%)<br>5(12,5%)	1(3,7%)<br>14(51,9%)<br>9(33,3%)<br>3(11,1%)	9(14,3%)<br>21(33,3%)<br>27(42,9%)<br>6(9,5%)	4(5,3%)<br>34(45,3%)<br>30(40,0%)<br>7(9,3%)	0,254	*no*<br>*yes*	27(67,5%)<br>13(32,5%)	23(85,2%)<br>4(14,8%)	50(79,4%)<br>13(20,6%)	68(90,7%)<br>7(9,3%)	**0,025**
dependence on machines´ speed	*never*<br>*rarely*<br>*moderately*<br>*often*	16(40,0%)<br>20(50,0%)<br>4(10,0%)<br>0(0,0%)	11(40,7%)<br>15(55,6%)<br>1(3,7%)<br>0(0,0%)	35(55,6%)<br>22(34,9%)<br>4(6,3%)<br>2(3,2%)	43(57,3%)<br>27(36,0%)<br>4(5,3%)<br>1(1,3%)	0,117	*no*<br>*yes*	39(97,5%)<br>1(2,5%)	26(96,3%)<br>1(3,7%)	58(92,1%)<br>5(7,9%)	73(97,3%)<br>2(2,7%)	0,597
give instructions	*never*<br>*rarely*<br>*moderately*<br>*often*	0(0,0%)<br>16(40,0%)<br>17(42,5%)<br>7(17,5%)	2(7,4%)<br>13(48,1%)<br>12(44,4%)<br>0(0,0%)	1(1,6%)<br>13(20,6%)<br>34(54,0%)<br>15(23,8%)	4(5,3%)<br>22(29,3%)<br>41(54,7%)<br>8(10,7%)	**0,014**	*no*<br>*yes*	30(75,0%)<br>10(25,0%)	21(77,8%)<br>6(22,2%)	48(76,2%)<br>15(23,8%)	69(92,0%)<br>6(8,0%)	**0,029**
responsibility for machines, material	*never*<br>*rarely*<br>*moderately*<br>*often*	0(0,0%)<br>9(22,5%)<br>18(45,0%)<br>13(32,5%)	0(0,0%)<br>13(48,1%)<br>8(29,6%)<br>6(22,2%)	5(7,9%)<br>4(6,3%)<br>28(44,4%)<br>26(41,3%)	4(5,3%)<br>9(12,0%)<br>35(46,7%)<br>27(36,0%)	**<0,001**	*no*<br>*yes*	28(70,0%)<br>12(30,0%)	23(85,2%)<br>4(14,8%)	51(81,0%)<br>12(19,0%)	66(88,0%)<br>9(12,0%)	0,138

2c. Most frequent stress factors originating from work environment and their evaluation

	"How often or how strongly does this characteristic or factor apply to your work?"					p_{χ^2} Pearson	"Does this make you feel physically or mentally stressed or strained?"					p_{χ^2} Pearson
stress factor		A	B	G	S			A	B	G	S	
warmth/heat	*never*	0(0,0%)	0(0,0%)	3(4,8%)	2(2,7%)	0,538	*no*	16(40,0%)	14(51,9%)	40(63,5%)	50(66,7%)	0,065
	rarely	16(40,0%)	6(22,2%)	23(36,5%)	27(36,0%)		*yes*	24(60,0%)	13(48,1%)	23(36,5%)	25(33,3%)	
	moderately	14(35,0%)	15(55,6%)	30(47,6%)	34(45,3%)							
	often	10(25,0%)	6(22,2%)	7(11,1%)	12(16,0%)							
improper lighting	*never*	1(2,5%)	0(0,0%)	5(7,9%)	4(5,3%)	0,476	*no*	17(42,5%)	17(63,0%)	38(60,3%)	51(68,0%)	0,125
	rarely	18(45,0%)	8(29,6%)	18(28,6%)	27(36,0%)		*yes*	23(57,5%)	10(37,0%)	25(39,7%)	24(32,0%)	
	moderately	14(35,0%)	16(59,3%)	30(47,6%)	32(42,7%)							
	often	7(17,5%)	3(11,1%)	10(15,9%)	12(16,0%)							
risk of accidents	*never*	0(0,0%)	0(0,0%)	2(3,2%)	2(2,7%)	0,768	*no*	22(55,0%)	16(59,3%)	39(61,9%)	49(65,3%)	0,770
	rarely	11(27,5%)	10(37,0%)	16(25,4%)	15(20,0%)		*yes*	18(45,0%)	11(40,7%)	24(38,1%)	26(34,7%)	
	moderately	14(35,0%)	8(29,6%)	24(38,1%)	35(46,7%)							
	often	15(37,5%)	9(33,3%)	21(33,3%)	23(30,7%)							
wetness, humidity	*never*	0(0,0%)	0(0,0%)	6(9,5%)	3(4,0%)	0,088	*no*	19(47,5%)	14(51,9%)	44(69,8%)	56(74,7%)	**0,025**
	rarely	19(47,5%)	11(40,7%)	24(38,1%)	40(53,3%)		*yes*	21(52,5%)	13(48,1%)	19(30,2%)	19(25,3%)	
	moderately	14(35,0%)	10(37,0%)	29(46,0%)	23(30,7%)							
	often	7(17,5%)	6(22,2%)	4(6,3%)	9(12,0%)							
odors, steam	*never*	0(0,0%)	0(0,0%)	3(4,8%)	5(6,7%)	0,439	*no*	18(45,0%)	16(59,3%)	42(66,7%)	54(72,0%)	**0,005**
	rarely	17(42,5%)	13(48,1%)	21(33,3%)	30(40,0%)		*yes*	22(55,0%)	11(40,7%)	21(33,3%)	21(28,0%)	
	moderately	12(30,0%)	8(29,6%)	25(39,7%)	31(41,3%)							
	often	11(27,5%)	6(22,2%)	14(22,2%)	9(12,0%)							
noise	*never*	0(0,0%)	0(0,0%)	5(7,9%)	3(4,0%)	0,387	*no*	22(55,0%)	16(59,3%)	41(66,1%)	53(70,7%)	0,154
	rarely	18(45,0%)	12(44,4%)	24(38,1%)	31(41,3%)		*yes*	18(45,0%)	11(40,7%)	21(33,9%)	22(29,3%)	
	moderately	16(40,0%)	9(33,3%)	21(33,3%)	31(41,3%)							
	often	6(15,0%)	6(22,2%)	13(20,6%)	10(13,3%)							
draft	*never*	1(2,5%)	0(0,0%)	8(12,7%)	8(10,7%)	0,496	*no*	24(60,0%)	17(63,0%)	47(74,6%)	60(80,0%)	0,154
	rarely	23(57,5%)	12(44,4%)	30(74,6%)	37(49,3%)		*yes*	16(40,0%)	10(37,0%)	16(25,4%)	15(20,0%)	
	moderately	11(27,5%)	13(48,1%)	21(33,3%)	24(32,0%)							
	often	5(12,5%)	2(7,4%)	4(6,3%)	6(8,0%)							
chemical substances	*never*	1(2,5%)	0(0,0%)	8(12,7%)	7(9,3%)	**0,001**	*no*	22(55,0%)	16(59,3%)	49(77,8%)	59(78,7%)	**0,015**
	rarely	15(37,5%)	19(70,4%)	38(60,3%)	44(58,7%)		*yes*	18(45,0%)	11(40,7%)	14(22,2%)	16(21,3%)	
	moderately	16(40,0%)	3(11,1%)	13(20,6%)	18(24,0%)							
	often	8(20,0%)	5(18,5%)	4(6,3%)	6(8,0%)							
dust, dirt	*never*	2(5,0%)	0(0,0%)	8(12,7%)	7(9,3%)	0,395	*no*	22(55,0%)	16(59,3%)	51(81,0%)	60(80,0%)	**0,003**
	rarely	17(42,5%)	16(59,3%)	25(39,7%)	41(54,7%)		*yes*	18(45,0%)	11(40,7%)	12(19,0%)	15(20,0%)	
	moderately	14(35,0%)	7(25,9%)	23(36,5%)	21(28,0%)							
	often	7(17,5%)	4(14,8%)	7(11,1%)	6(8,0%)							
