# Supplementary material for: Subjective perceptions of workload and stress of emergency service personnel depending on work-related behavior and experience patterns
Source: Notf Rett Med. 2022 Sep 8;25(Suppl 2):15–22. doi: 10.1007/s10049-022-01076-y (PMC9454386; doi:10.1007/s10049-022-01076-y)
Supplement: Supplementary file 3 — ESM 3: Physical, psychological, and social-communicative impairments within the AVEM groups [file 10049_2022_1076_MOESM3_ESM.pdf]

Supplementary material to the article "***Subjective perceptions of workload and stress of emergency service personnel depending on work-related behavior and experience patterns***" by Beatrice Thielmann, Heiko Schumann, Julia Botscharow and Irina Böckelmann (2022) in Notfall+Rettungsmdedizin.
Article and supplementary material are available at www.springermedizin.de. Please enter the article title in the search there

3: Physical, psychological and social-communicative impairments within the AVEM groups.

**KOEPS dimensions**	**AVEM sample**				**PKruskal - Valais**	**P Mann -Whitney**
	**A**	**B**	**G**	**S**		
	AV ± SD<br>Median (Min - Max)<br>[95% - AI]					
**KOEPS**						
**Physical impairment**	5,6 ± 1,43<br>5,5 (3 - 9)<br>[5,2 - 6,1]	5,6 ± 1,45<br>6 (3 - 8)<br>[5,1 - 6,2]	4,2 ± 1,59<br>4 (2 - 9)<br>[3,8 - 4,6]	4,1 ± 1,55<br>4 (2 - 8)<br>[3,7 - 4,4]	**<0,001**	A-G***<br>A-S***<br>B-G***<br>B-S***
**Mental impairment**	5,6 ± 1,32<br>5 (4 - 8)<br>[5,1 - 6,0]	6,0 ± 1,72<br>6 (3 - 9)<br>[5,4 - 6,7]	4,1 ± 1,55<br>4 (2 - 9)<br>[3,7 - 4,4]	3,8 ± 1,43<br>4 (1 - 8)<br>[3,5 - 4,2]	**<0,001**	A-G***<br>A-S***<br>B-G***<br>B-S***
**Social-communicative Impairment**	5,0 ± 1,25<br>5 (3 - 8)<br>[4,6 - 5,4]	5,6 ± 1,62<br>5 (3 - 9)<br>[5,0 - 6,2]	3,8 ± 1,12<br>3 (3 - 8)<br>[3,5 - 4,1]	3,8 ± 1,10<br>3 (3 - 8)<br>[3,6 - 4,1]	**<0,001**	A-G***<br>A-S***<br>B-G***<br>B-S***
**Total**	5,4 ± 1,36<br>5 (4 - 9)<br>[5,0 - 5,9]	5,9 ± 1,76<br>6 (3 - 9)<br>[5,2 - 6,6]	3,7 ± 1,65<br>4 (1 - 9)<br>[3,3 - 4,1]	3,5 ± 1,50<br>4 (1 - 8)<br>[3,3 - 4,1]	**<0,001**	A-G***<br>A-S***<br>B-G***<br>B-S***
Notes. *** p<0.001. CI=confidence interval, AV ± SD= average valvue and standard deviation.						
