# Supplementary material for: Subjective perceptions of workload and stress of emergency service personnel depending on work-related behavior and experience patterns
Source: Notf Rett Med. 2022 Sep 8;25(Suppl 2):15–22. doi: 10.1007/s10049-022-01076-y (PMC9454386; doi:10.1007/s10049-022-01076-y)
Supplement: Supplementary file 4 — ESM 4: Correlation analysis according to Spearman of the AVEM dimensions with the main scales of the KOEPS and EBF [file 10049_2022_1076_MOESM4_ESM.pdf]

***Supplementary material** to the article "**Subjective perceptions of workload and stress of emergency service personnel depending on work-related behavior and experience patterns**" by Beatrice Thielmann, Heiko Schumann, Julia Botscharow and Irina Böckelmann (2022) in Notfall+Rettungsmdedizin.*
Article

4: Correlation analysis according to Spearman of the AVEM dimensions with the main scales of the KOEPS and EBF.

		AVEM										
		BA	BE	VB	PS	DF	RT	OP	IR	EE	LZ	SU
KOEPS	**G**		0,180**	0,341***		-0,400***	0,396***		-0,305***	-0,282***	-0,404***	-0,223***
EBF	**BEA**			0,308***		-0,417***	0,370***	-0,147*	-0,322***	-0,262***	-0,468***	-0,251***
	**ERH**			-0,251***		0,498***	-0,333***	0,253***	0,334***	0,343***	0,532***	0,308***

Notes. * p< 0.05; ** p < 0.01; *** p < 0.001.
Abbreviations for AVEM dimensions*: BA: Subjective importance of work, BE: Work-related ambition, VB: Willingness to work until exhausted, PS: Striving for perfection, DF: Distancing ability, RT: Tendency to resignation in the face of failure, OP: Proactive problem-solving, IR: Inner calm and balance, EE: Experience of success at work, LZ: Satisfaction with life, SU: Experience of social support.* Abbreviations for KOEPS scales: *G - Total impairments.* Abbreviations for EBF subscales and main scales: *BEA – strain. ERH – Recovery.*
